# A Universal Method for Identifying and Correcting Induced Heave Error in Multi-Beam Bathymetric Surveys

**DOI:** 10.3390/s26020618

**Published:** 2026-01-16

**Authors:** Xiaohan Yu, Yang Cui, Jintao Feng, Shaohua Jin, Na Chen, Yuan Wei

**Affiliations:** Department of Oceanography and Hydrography, Dalian Naval Academy, Dalian 116018, China; yuxiaohandljy@163.com (X.Y.); 13998435151@163.com (Y.C.); jsh_1978@163.com (S.J.); 13478521431@163.com (N.C.); weiyuan940324@163.com (Y.W.)

**Keywords:** multibeam, induced heave, regression diagnostics, Partial Least Squares Regression

## Abstract

Addressing the difficulty of intuitively identifying and effectively correcting induced heave error in multibeam measurements, this paper proposes a two-stage methodology comprising error identification and correction. This scheme includes an error discrimination method based on regression diagnostics and an error correction method based on Partial Least Squares Regression (PLSR). By establishing a mathematical model between bathymetric discrepancies and attitude parameters, statistical diagnosis and effective identification of the error are achieved. To further mitigate the impact of induced heave error on bathymetric data, an elimination model based on PLSR is developed, enabling high-precision prediction and compensation of the induced heave error. Validation using field survey data demonstrates that this method can effectively estimate the installation offset parameters of the attitude sensor. After correction, the root mean square of bathymetric discrepancies between adjacent survey lines is reduced by approximately 78.8%, periodic stripe-shaped distortions along the track direction are essentially eliminated, and the quality of terrain mosaicking is significantly improved. This provides an effective solution for controlling induced heave error under complex topographic conditions.

## 1. Introduction

The Multibeam Echo Sounder (MBES), renowned for its wide coverage, high efficiency, and high-resolution capabilities, has revolutionized the operational paradigm of traditional single-beam sounding and become a primary technique for acquiring high-resolution seabed topographic data [1,2]. Induced heave error, resulting from the installation deviation of attitude sensors, is one of the key factors affecting the quality of multibeam bathymetric data [3]. When the actual installation position of an attitude sensor deviates from its theoretically designed location, the pitch and yaw motions of the vessel will, through the lever arm effect, cause the transducer to generate additional vertical heave error, known as induced heave error. This error typically manifests as periodic stripe-like distortions along the track direction in bathymetric products, significantly degrading the accuracy and usability of seabed topographic data.

Currently, compensation for induced heave error often relies on precise calibration during the sensor installation phase. However, in practical operations, residual installation biases are difficult to completely avoid due to complex working conditions, imperfect calibration, or equipment displacement, leading to induced heave error systematically affecting sounding results [4]. Particularly in areas with complex topography, the morphological similarity between natural seabed variations (e.g., sand waves, valleys) and distortions caused by induced heave error makes effective distinction challenging through visual interpretation or empirical analysis alone, thereby increasing the difficulty of error identification and correction.

Regarding the elimination of anomalous stripes caused by induced heave error, two main technical approaches have been developed: The first is filtering methods based on signal processing, such as trend surface filtering and frequency-domain filtering, which aim to separate the error signal from the true terrain by constructing background trend surfaces or designing filters. For instance, Huang et al. [5] proposed using a Least Squares Support Vector Machine model to construct a terrain trend surface for filtering, showing some suppression effect on stripes induced by heave error. Dong et al. [6] proposed a bathymetric data filtering method based on the rolling circle transform, processing multibeam data Ping by Ping; however, this method is primarily suitable for flat areas and has limited effectiveness in complex terrains. Zhang et al. [7] proposed an improved trend surface filtering algorithm for multibeam sounding data based on the local minimum range of scattered sounding points, which gradually filters outliers through local surface fitting and better preserves true seabed features, but its effectiveness in mitigating regular stripe errors is not significant. Zhang et al. [8] introduced median filtering before trend surface filtering to perform weighted correction on depth data, enhancing the algorithm’s robustness, yet like other filtering methods, it still does not achieve ideal results in eliminating systematic stripes along the track. These methods are straightforward to implement and computationally efficient, offering good smoothing effects for isolated outliers and slight stripe noise in flat or gently sloping terrain. However, they also have notable limitations. Firstly, the filtering effectiveness heavily depends on the selection of polynomial order and local window size; inappropriate parameters can lead to under-filtering or over-smoothing. Secondly, in areas with complex and rugged topography, low-order polynomials struggle to accurately fit the real terrain, potentially misidentifying normal steep features as outliers or failing to effectively detect stripe errors superimposed on complex topography.

The second approach is correction methods based on error modeling, which involve establishing a mathematical model between attitude parameters and bathymetric discrepancies to directly estimate the sensor installation bias parameters, thereby eliminating the error influence from the raw data. For example, Long et al. [9] proposed a method for the quadratic detection of attitude sensor position bias based on depth discrepancies between corresponding soundings from reciprocal survey lines. By modeling the relationship between sensor position bias and depth discrepancies and using support vector regression to estimate the position bias, they effectively removed the influence of induced heave error from the bathymetric data. This type of method can fundamentally eliminate systematic errors, and its correction results have clear physical significance, maximizing the retention of true seabed topographic details and avoiding the potential terrain blurring associated with filtering methods. However, the challenge lies in the accurate diagnosis of the error source and the precise establishment of the error model, requiring considerable operator experience and data analysis skills.

Based on an analysis of the mechanism generating induced heave error, this paper systematically investigates its discrimination and correction methods. Firstly, an error discrimination method based on regression diagnostics is proposed. This involves establishing a multiple linear regression model between bathymetric discrepancies and attitude parameters, combined with significance testing and residual analysis, to achieve objective identification of the error source. Furthermore, addressing the instability of traditional least squares estimation under multicollinearity conditions, the Partial Least Squares Regression (PLSR) method is introduced to construct a high-precision correction model for induced heave error, providing effective technical support for enhancing the quality of multibeam bathymetric data.

## 2. Method for Identifying Induced Heave Error

In complex topographic areas, the natural undulating features of the seabed (such as sand waves, ripples, grooves, etc.) are highly similar in morphology to the distortions caused by induced heave error, leading to easy confusion between the two in the data [10]. Since sand wave topography itself exhibits periodic fluctuations, its spatial distribution characteristics may overlap in spectrum and spatial scale with the wavy depth offsets caused by induced heave error [11]. Relying solely on subjective interpretation or empirical analysis makes it difficult to effectively distinguish between them. Therefore, there is an urgent need to construct an objective discrimination method based on mathematical modeling and statistical diagnosis. By quantitatively analyzing the correlation between bathymetric discrepancies and attitude parameters, the characteristics of residual distribution, and the laws of error propagation, the coupling effect between natural topographic variation and error distortion can be decoupled, thereby enabling accurate identification and correction of induced heave error in complex scenarios. This paper proposes a heave error discrimination method based on regression diagnostics [12]. It establishes a mathematical relationship between bathymetric discrepancies and attitude parameters using a linear regression model and systematically identifies error sources by comprehensively applying parameter significance testing, goodness-of-fit analysis, and residual distribution verification.

### 2.1. Impact of Induced Heave Error on Sounding Point Position

**Figure 1:**
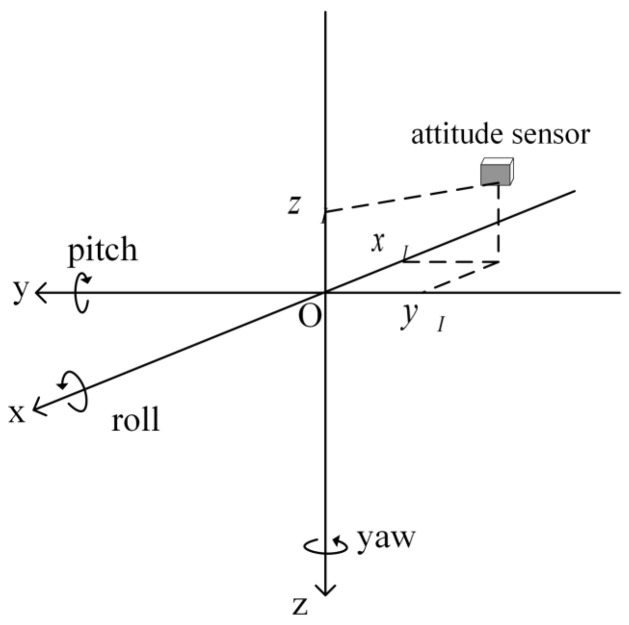
Positional relationship between the attitude sensor and the transducer.

During operations, the survey vessel is affected by swells, leading to periodic heave motion, which can be monitored by the attitude sensor. Due to roll and pitch motions, the vessel cannot maintain a perfectly horizontal state, resulting in differences in heave motion at different locations on the vessel. This phenomenon of inconsistent heave between the transducer and the attitude sensor caused by roll and pitch is termed induced heave. The positional relationship between the attitude sensor and the transducer is shown in Figure 1.

In Figure 1, the origin of the coordinate system is the center of the transducer, the *x*-axis points towards the bow, the *y*-axis points towards the starboard side, and the *z*-axis points towards the seabed. The roll angle R is defined as positive when the starboard side pitches downward, and the pitch angle P is positive when the bow pitches upward. The induced heave $h_{i}$ can be calculated from the offset of the attitude sensor relative to the transducer $\left(x_{I},y_{I},z_{I}\right)$ and the roll angles R and pitch angles P provided by the attitude sensor at different times using the following formula [13]:

(1) $$h_{i}=x_{I}\sin P-y_{I}\sin R\cos P-z_{I}(\cos R\cos P-1)$$

The heave of the transducer is the algebraic sum of the measured heave and the induced heave:

(2) $$h_{s}=h_{i}+h_{m}$$

The heave of the transducer affects the depth of the sounding point. From Equation (2), it can be seen that when errors exist in the measured heave and the induced heave, they will cause depth deviation $\Delta z_{heave}$ in the sounding point, with a value of:

(3) $$\Delta z_{heave}=\Delta h_{i}+\Delta h_{m},$$

where $\Delta h_{m}$ is the measurement error of the heave measured by the attitude sensor, and $\Delta h_{i}$ is the induced heave error resulting from the positional offset of the attitude sensor relative to the transducer and the measurement errors in the roll and pitch angles provided by the attitude sensor. Its value is:

(4) $$\Delta h_{i}=({\tilde{x}}_{I}-x_{I}^{\prime })\sin P-({\tilde{y}}_{I}-y_{I}^{\prime })\sin R\cos P-({\tilde{z}}_{I}-z_{I}^{\prime })(\cos R\cos P-1),$$

where ${\tilde{x}}_{I},{\tilde{y}}_{I},{\tilde{z}}_{I}$ represent the true value of the attitude sensor positional offset, and $x_{I}^{\prime },y_{I}^{\prime },z_{I}^{\prime }$ are the observed value containing measurement errors.

### 2.2. Induced Heave Error Discrimination Method Based on Regression Diagnostics

#### 2.2.1. Establishment of the Induced Heave Error Regression Model

Assuming that only induced heave error is present in the survey lines, the depth values of a corresponding point pair at the same location in two adjacent lines, a and b, $z_{a},z_{b}$ are, respectively:

(5) $$\left\{\begin{array}{l} z_{a}=\tilde{z}+\Delta h_{ia}+\varepsilon_{a}\\ z_{b}=\tilde{z}+\Delta h_{ib}+\varepsilon_{b} \end{array}\right.,$$

where $\tilde{z}$ is the true water depth at that location, and $\Delta h_{ia},\Delta h_{ib}$ are the induced heave errors contained in the depth values of the corresponding point pair, and $\varepsilon_{a},\varepsilon_{b}$ are the residual error. Taking the difference of the depth values for this corresponding point pair and combining it with Equation (4) yields the bathymetric discrepancy dz:

(6) $$\begin{array}{l} dz=z_{a}-z_{b}=(\Delta h_{ia}-\Delta h_{ib})+(\varepsilon_{a}-\varepsilon_{b})\\ \;=(({\tilde{x}}_{I}-x_{I}^{\prime })(\sin P_{a}-\sin P_{b})-({\tilde{y}}_{I}-y_{I}^{\prime })\\ \;(\cos P_{a}\sin R_{a}-\cos P_{b}\sin R_{b})-({\tilde{z}}_{I}-z_{I}^{\prime })\\ \;(\cos R_{a}\cos P_{a}-\cos R_{b}\cos P_{b}))+d\varepsilon \;, \end{array}$$

where ${\tilde{x}}_{I},{\tilde{y}}_{I},{\tilde{z}}_{I}$ are the true value of the attitude sensor positional offset, $x_{I}^{\prime },y_{I}^{\prime },z_{I}^{\prime }$ are the observed value containing measurement errors, and dε is the difference in residual errors, which should follow a normal distribution. To simplify the expression, three explanatory variables X,Y,Z are defined:

(7) $$\begin{array}{l} X=\sin P_{a}-\sin P_{b}\\ Y=\cos P_{a}\sin R_{a}-\cos P_{b}\sin R_{b}\\ Z=\cos R_{a}\cos P_{a}-\cos R_{b}\cos P_{b} \end{array}$$

Analyzing Equation (6) shows that when induced heave error is present in the survey lines, the bathymetric discrepancy between corresponding point pairs in the two lines satisfies a linear relationship with X,Y,Z; conversely, if no induced heave error exists, the linear relationship between them is not significant.

Based on the effective matching distance R, corresponding point pairs between two adjacent survey lines are extracted. The value of R is determined by the average point cloud density within the overlapping area of the main survey line and the check line, specifically defined as:

(8) $$R=\frac{num}{S},$$

where num is the number of multibeam sounding points in the overlapping area, and S is the area of the overlapping region between the main survey line and the check line. Since factors such as sound velocity errors, vessel attitude, and installation biases can affect the horizontal position of sounding points, these errors must be preprocessed to ensure that the extracted corresponding point pairs indeed represent the same location on the seabed. After extracting the corresponding point pairs, calculate dz,X,Y,Z for each pair to fit the regression model. It is particularly important to note that the selected adjacent survey lines should be as parallel as possible and maintain a stable heading; otherwise, systematic errors related to the incidence angle might be introduced into the bathymetric discrepancies, affecting the normality of the regression model residuals.

Subsequently, based on Equation (6), a multiple linear regression model is established:

(9) $$dz=\beta_{0}+\beta_{1}X+\beta_{2}Y+\beta_{3}Z+d\varepsilon ,$$

where $\beta_{0}$ is the intercept term, reflecting the combined influence of other residual systematic errors, and $\beta_{1},\beta_{2},\beta_{3}$ respectively represent the contribution coefficients of the independent variables X,Y,Z to the bathymetric discrepancy. The model parameters are estimated using the least squares method, yielding the regression model.

#### 2.2.2. Model Testing and Evaluation

After obtaining the regression model, its fitting performance is evaluated through significance testing and goodness-of-fit analysis [14].

1. Significance Testing

The statistical significance of the regression coefficients is assessed using the t-test. For each coefficient $\beta_{i}(i=1,2,3)$, assuming $\beta_{i}=0$, the t-statistic is constructed:

(10) $$t=\frac{{\hat{\beta }}_{i}}{SE({\hat{\beta }}_{i})},$$

where ${\hat{\beta }}_{i}$ is the estimated value of the coefficient $\beta_{i}$, and $SE({\hat{\beta }}_{i})$ is the standard deviation of the regression coefficient. Under the null hypothesis $\beta_{i}=0$, the t-statistic follows a t-distribution with degrees of freedom n−k−1 (where n is the sample size, and k is the number of independent variables). If the calculated p-value is less than the significance level (α=0.05), the null hypothesis is rejected, indicating that the influence of the corresponding independent variable on the bathymetric discrepancy is statistically significant, thereby excluding random noise interference. When the p-value for all coefficient t-tests are less than 0.05, it can be considered that there is a possibility of a linear relationship between dz and X,Y,Z, otherwise, the linear relationship between the variables should be negated.

2. Goodness-of-Fit Analysis

The explanatory power of the model is evaluated using the coefficient of determination $R^{2}$ and the adjusted coefficient of determination $adjR^{2}$. $R^{2}$ is defined as the proportion of the variance explained by the model to the total variance:

(11) $$R^{2}=1-\frac{RSS}{TSS},$$

where $RSS={\sum \left(dz-\hat{dz}\right)}^{2}$ is the residual sum of squares, and $TSS={\sum \left(dz-\bar{dz}\right)}^{2}$ is the total sum of squares. The closer $R^{2}$ is to 1, the more of the variation in bathymetric discrepancies can be explained by the attitude parameters. However, $R^{2}$ increases monotonically with the addition of independent variables, potentially masking the risk of overfitting. To address this, the adjusted coefficient of determination $adjR^{2}$ is introduced:

(12) $$adjR^{2}=1-\frac{RSS/(n-k-1)}{TSS/(n-1)}$$

It penalizes the number of independent variables, providing a more robust reflection of the model’s true explanatory power. This study considers that when $adjR^{2}>0.6$, the model can effectively capture the linear relationship between the variables.

#### 2.2.3. Residual Analysis and Assumption Verification

The reliability of the regression model depends on the statistical assumptions about the error term (homoscedasticity, normality), which must be strictly verified through residual analysis.

1. Residuals vs. Fitted Values Plot

A scatter plot is drawn with the model predicted values $\hat{dz}$ on the horizontal axis and the residuals $dz-\hat{dz}$ on the vertical axis. If the residuals are randomly distributed around the zero line without any discernible trend, the homoscedasticity assumption is satisfied, indicating that the model correctly captures the linear relationship between the variables. If the residuals exhibit a curved trend (such as U-shaped or parabolic), it suggests a violation of the homoscedasticity assumption. This can lead to misestimation of the standard errors of the regression coefficients, rendering confidence intervals invalid and significance tests ineffective.

2. Residual Quantile–Quantile (Q-Q) Plot

The normality of residuals is a core assumption for the validity of the linear regression model. The residual Q-Q (Quantile–Quantile) plot is used to check whether the residuals follow a normal distribution [15]. It compares the quantiles of the sample data against the quantiles of a theoretical normal distribution. If the points on the Q-Q plot approximately lie along a straight line, it indicates that the residuals approximately follow a normal distribution; if the points deviate from the line, it suggests skewness or other distributional characteristics.

3. Anderson–Darling Normality Test

The Anderson–Darling (A-D) normality test is a statistical method used to determine whether a dataset follows a normal distribution. The A-D test is employed for further quantitative analysis of the normality of the residuals [16], providing the corresponding statistic $A^{2}$:

(13) $$A^{2}=-n-\frac{1}{n}\sum_{i=1}^{n}\left(2i-1)[\ln F(e_{\left(i\right)})+\ln(1-F(e_{\left(n+1-i\right)}))\right],$$

where F(⋅) is the cumulative distribution function of the standard normal distribution, and $e_{\left(i\right)}$ are the sorted residuals. If p<0.05 corresponding to the A-D statistic is less than the significance level, the normality hypothesis should be rejected, deeming the specified linear model unreasonable; conversely, it indicates that the residuals strictly conform to a normal distribution, and the model assumption is reasonable.

Finally, if the model passes the significance tests and the residuals satisfy the homoscedasticity and normality assumptions, it can be concluded that the heave error indeed exists.

## 3. Method for Correcting Induced Heave Error

**Figure 2:**
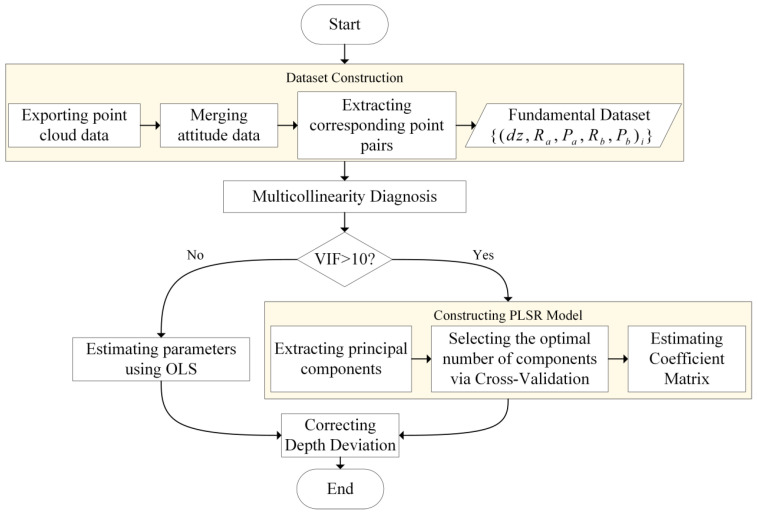
Flowchart of induced heave error correction.

When it is confirmed that the anomalous topographic stripes are caused by induced heave error, a secondary calibration of the installation position offset of the attitude sensor is necessary to mitigate the impact of this error on the topographic data. To this end, this paper proposes an induced heave error elimination method based on PLSR, which mainly includes four steps: dataset construction, multicollinearity diagnosis, parameter estimation, and depth deviation correction. The workflow is shown in Figure 2. The method is elaborated in detail below.

### 3.1. Dataset Construction

**Figure 3:**
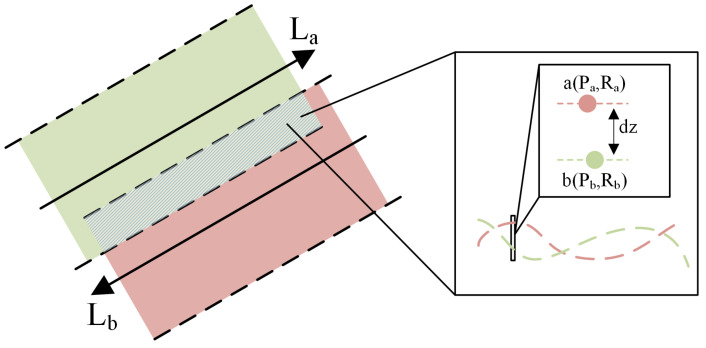
Schematic diagram of the overlapping area of adjacent survey lines.

To build the induced heave error correction model, it is first necessary to acquire the fundamental dataset used for modeling. The core concept of the method proposed in this paper is to invert the installation offset parameters of the attitude sensor using the bathymetric discrepancies in the overlapping areas of adjacent survey lines [17]. The required data include the bathymetric discrepancies dz, as well as the roll angles $R_{a},R_{b}$ and pitch angles $P_{a},P_{b}$ from the attitude sensor corresponding to each sounding point, as shown in Figure 3. The data acquisition and processing workflow is as follows:

First, software such as CARIS HIPS 12.1 is used to perform preprocessing on the raw multibeam bathymetric data, including sound velocity correction, tidal correction, and outlier filtering [18]. The measurement data from two adjacent survey lines are exported, including the latitude and longitude coordinates, depth values, and corresponding timestamps of each sounding point. To effectively separate the induced heave error from bathymetric data affected by multiple error sources, interference from other error factors must be suppressed as much as possible. Considering that angle-dependent errors like sound velocity errors and installation biases significantly affect the positional accuracy of sounding points, only central beam sounding points with beam incidence angles between −30° and 30° are used for modeling [19].

Subsequently, the roll and pitch observations from the attitude sensor records at the corresponding time series are extracted. The bathymetric data and attitude data are matched based on the measurement timestamps, assigning the roll and pitch angles at the corresponding time to each sounding point. Then, the latitude and longitude coordinates of the sounding points are uniformly converted to UTM projected coordinates to facilitate planar distance calculations [20].

On this basis, corresponding point pairs meeting the criteria within the overlapping area of the two survey lines are extracted according to the effective matching distance, R. Finally, p sets of valid corresponding point pairs are obtained, forming the fundamental dataset $\left\{{\left(dz,R_{a},P_{a},R_{b},P_{b}\right)}_{i},i=1,\cdots \cdots ,p\right\}$ for PLSR modeling. This dataset will provide the basis for subsequent multicollinearity diagnosis and parameter estimation.

### 3.2. Multicollinearity Diagnosis

Substituting the fundamental dataset into the regression model Equation (9) yields:

(14) $$\left\{\begin{array}{c} dz_{1}=\beta_{0}+\beta_{1}X_{1}+\beta_{2}Y_{1}+\beta_{3}Z_{1}+d\varepsilon_{1}\\ dz_{2}=\beta_{0}+\beta_{1}X_{2}+\beta_{2}Y_{2}+\beta_{3}Z_{2}+d\varepsilon_{2}\\ \vdots \\ dz_{p}=\beta_{0}+\beta_{1}X_{p}+\beta_{2}Y_{p}+\beta_{3}Z_{p}+d\varepsilon_{p} \end{array}\right.$$

The matrix form of Equation (14) is

(15) $$Y_{0}=X_{0}A+\xi ,$$

where $Y_{0}={\left[\begin{array}{cccc} dz_{1} & dz_{2} & \cdots & dz_{p} \end{array}\right]}^{T}$ is the dependent variable matrix of the model,

$$X_{0}=\left[\begin{array}{cccc} 1 & X_{1} & Y_{1} & Z_{1}\\ 1 & X_{2} & Y_{2} & Z_{2}\\ \vdots & \vdots & \vdots & \vdots \\ 1 & X_{p} & Y_{p} & Z_{p} \end{array}\right]$$

is the independent variable matrix of the model, $A={\left[\begin{array}{cccc} \beta_{0} & \beta_{1} & \beta_{2} & \beta_{3} \end{array}\right]}^{T}$ is the coefficient matrix, and $\xi ={\left[\begin{array}{cccc} d\varepsilon_{1} & d\varepsilon_{2} & \cdots & d\varepsilon_{p} \end{array}\right]}^{T}$ is the residual vector.

For Equation (15), the least squares estimate of the coefficient matrix A should be [21]:

(16) $${\hat{A}}_{OLS}={\left(X^{T}X\right)}^{-1}X^{T}Y$$

However, since the independent variables are all composed of trigonometric functions of the attitude angles, there may be strong multicollinearity among them. Multicollinearity causes the design matrix $X^{T}X$ to tend towards singularity, thereby significantly increasing the diagonal elements of its inverse matrix (corresponding to the variance of the parameter estimates ${\hat{A}}_{OLS}$) and ultimately severely undermining the accuracy of parameter estimation.

The Variance Inflation Factor (VIF) is an indicator measuring the degree of multicollinearity in a regression model. Its calculation formula is:

(17) $$VIF={\left(1-R^{2}\right)}^{-1}$$

where $R^{2}$ is the coefficient of determination obtained by regressing one independent variable against the others. Calculate the VIF for each independent variable sequentially and use the maximum value among them as the basis for multicollinearity diagnosis. $VIF_{\max}=1$ indicates complete independence among variables, $1<VIF_{\max}<5$ suggests mild multicollinearity, $5\leq VIF_{\max}<10$ indicates moderate multicollinearity, and $VIF_{\max}>10$ signifies severe multicollinearity [22]. In such cases, the estimates obtained by traditional least squares regression are unreliable. The following section will employ the PLSR model to estimate the coefficient matrix A [23].

### 3.3. PLSR Model Construction

PLSR is a multivariate data analysis method in statistics and machine learning, particularly suitable for situations where multicollinearity exists between dependent and independent variables [24]. Its basic idea is to extract latent components from both the independent variable matrix X and the dependent variable matrix Y, such that these components not only summarize the information of the original variables well but also maximize the explanation of the dependent variable. The method is detailed below.

For Equation (15), extract the first component $t_{1}$ as:

(18) $${\hat{t}}_{1}=\left[\begin{array}{cccc} 1 & X_{1} & Y_{1} & Z_{1}\\ 1 & X_{2} & Y_{2} & Z_{2}\\ \vdots & \vdots & \vdots & \vdots \\ 1 & X_{p} & Y_{p} & Z_{p} \end{array}\right]\left[\begin{array}{c} \omega_{10}\\ \omega_{11}\\ \omega_{12}\\ \omega_{13} \end{array}\right]=X_{0}\omega_{1},$$

where $\omega_{1}$ is the eigenvector corresponding to the largest eigenvalue of the matrix $X_{0}^{T}Y_{0}Y_{0}^{T}X_{0}$. Establish the regression of $Y_{0},\;X_{0}$ onto $t_{1}$.

(19) $$\left\{\begin{array}{c} X_{0}=t_{1}\alpha_{1}^{T}+X_{1}\\ Y_{0}=t_{1}\beta_{1}^{T}+Y_{1} \end{array}\right.,$$

where $X_{1},\;Y_{1}$ are the residual matrix of $X_{0},\;Y_{0}$, and $\alpha_{1},\;\beta_{1}$ are the model effect load. The value of the model effect load c1 is obtained based on the least squares principle as:

(20) $$\left\{\begin{array}{c} \alpha_{1}=\frac{X_{0}^{T}t_{1}}{{\left\|t_{1}\right\|}^{2}}\\ \beta_{1}=\frac{Y_{0}^{T}{\hat{t}}_{1}}{{\left\|{\hat{t}}_{1}\right\|}^{2}} \end{array}\right.$$

Using $X_{1},\;Y_{1}$ instead of $X_{0},\;Y_{0}$, extract the second component $t_{2}$, and repeat the steps in Equations (18)–(20) until after extracting the r-th component, the residual matrix $Y_{r}$ of $Y_{0}$ is close to zero. At this point, we have:

(21) $$\left\{\begin{array}{c} X_{0}=t_{1}\alpha_{1}^{T}+\cdots +\\ Y_{0}=t_{1}\beta_{1}^{T}+\cdots + \end{array}\right.\begin{array}{c} t_{r}\alpha_{r}^{T}+X_{r}\\ t_{r}\beta_{r}^{T}+Y_{r} \end{array}$$

Substituting the score vectors of each component $t_{i}(i=1,2,\cdots \cdots ,r)$ into Equation (21) gives:

(22) $$Y_{0}=X_{0}WB+Y_{r},$$

where $W=(\omega_{1},\omega_{2},\cdots \cdots ,\omega_{r})$, $B={\left(\beta_{1},\beta_{2},\cdots \cdots ,\beta_{r}\right)}^{T}$. Comparing Equation (22) with Equation (15), the Partial Least Squares estimate of the coefficient matrix A can be obtained as:

(23) $${\hat{A}}_{\mathrm{PLS}}=WB$$

The selection of the number of components in PLSR has a significant impact on model performance. Too few components lead to underfitting, while too many may cause overfitting. This paper uses the k-fold cross-validation method to select the optimal number of components [25]. This method involves dividing the dataset into k subsets, repeatedly using k-1 subsets as the training set and the remaining one subset as the validation set, thereby evaluating the model’s generalization ability under different numbers of components.

The choice of the number of folds k in cross-validation requires a balance between bias and variance. When k is small, the training set size is relatively small, potentially leading to high estimation bias; when k is too large, the folds are highly correlated, leading to high estimation variance. Considering both computational efficiency and sample size, this paper adopts 5-fold cross-validation [26]. For each candidate number of components, the mean squared error of the 5-fold cross-validation is calculated, and the number of components that minimizes the cross-validation mean squared error is selected as the optimal number.

After obtaining the estimated value of the coefficient matrix A, combined with the attitude parameters R and P, the depth of multibeam sounding points can be corrected according to the following formula:

(24) $$z^{\prime }=z-[\beta_{1}\sin P-\beta_{2}\sin R\cos P-\beta_{3}(\cos R\cos P-1)],$$

where z and $z^{\prime }$ represent the water depth before and after correction, respectively. The performance of the above method will be verified and analyzed using field survey data in the following sections.

## 4. Experimental and Analysis

### 4.1. Trial Description

To verify the applicability and effectiveness of the proposed method under complex topographic conditions—where heave errors are more challenging to identify and correct compared to flat, deep-water areas—a test area with significant topographic relief was selected in the Yellow Sea, China. The region has an average water depth of about 20 m and features varied seabed morphology, providing a demanding environment for method validation. Two adjacent parallel survey lines, each approximately 2200 m in length, were surveyed using an EM2040C multibeam echo sounder system. The system was configured with a ping update rate of 27 Hz, operating in equidistant measurement mode with a sector opening angle of 110° and 256 beams per ping. To retain the original error characteristics for analysis, although the offset parameters of the attitude sensor were measured during the survey, real-time compensation was not applied during data acquisition.

**Figure 4:**
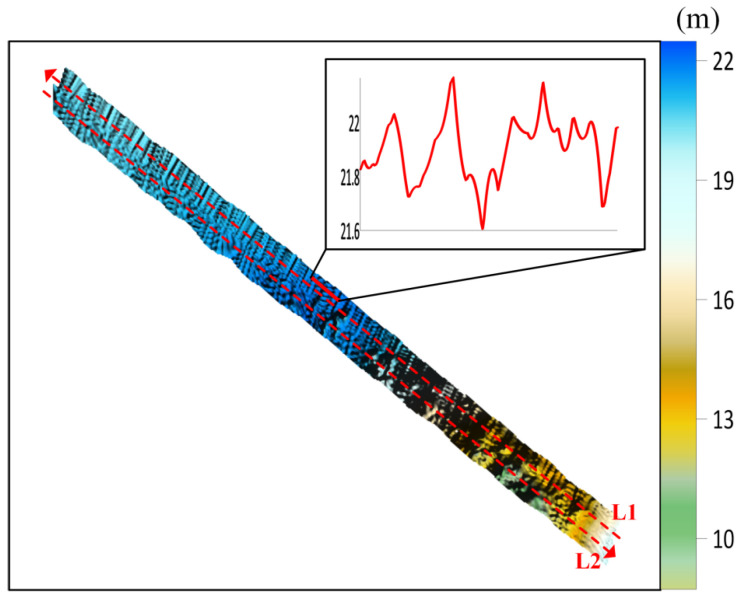
Measured topographic map.

The collected raw bathymetric data underwent quality control and position calculation. After removing abnormal sounding points and outer beam data, the multibeam point cloud data were obtained. After gross error removal and grid interpolation processing, the measured topographic map shown in Figure 4 was generated. Obvious stripe-shaped topographic distortions along the track direction can be observed in the figure, with significant fluctuations in local topographic profiles. Due to the inherently complex topographic variations in this area, the natural topographic features are morphologically similar to the distortions caused by induced heave error, making them difficult to distinguish effectively. Therefore, it is necessary to conduct the identification and correction of the induced heave error.

### 4.2. Validation of the Effectiveness of the Error Discrimination Method

**Figure 5:**
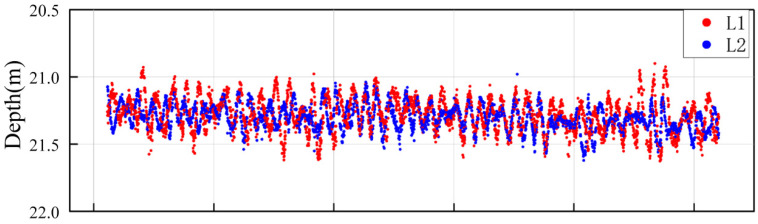
Partial point cloud profile used for regression diagnostics.

The induced heave error was discriminated according to the method described in Section 2. Bathymetric data from the same along-track profile of the two survey lines were extracted for linear regression model fitting. Based on the distance, corresponding point pairs were extracted, resulting in a total of 10,390 pairs. A partial point cloud profile is shown in Figure 5. The depth values of each corresponding point pair were integrated with the roll and pitch data of the survey vessel at the corresponding time, and the regression model was established according to Equation (9). The regression results and significance analysis results are detailed in Table 1. The results show that the p-value of the *t*-test for each independent variable are all less than the significance level of 0.05, indicating that the variables X, Y, Z have a statistically significant linear driving effect on the bathymetric discrepancy. Meanwhile, the calculated adjusted coefficient of determination $adjR^{2}$ for the model is 0.742, verifying the strong correlation between the heave error and the attitude parameters.

**Figure 6:**
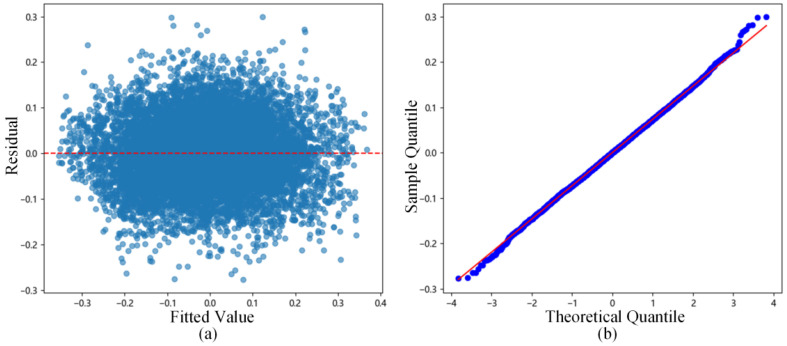
Residual analysis statistical chart for data containing induced heave error: (**a**) Residuals vs. fitted values plot; (**b**) Residual Q-Q plot.

The residuals of the fitting results were analyzed, and the residuals vs. fitted values plot (Figure 6a) and the residual Q-Q plot (Figure 6b) were drawn. Observing Figure 6a, the residuals are evenly distributed between −0.3 and 0.3, indicating homoscedasticity of the residuals. Observing Figure 6b, the points are basically distributed along the diagonal, supporting the assumption of residual normality. Simultaneously, the Anderson–Darling normality test was performed on the residuals, yielding a statistic $A^{2}$ value of 0.4270 and a p-value of 0.3131. This p-value is higher than the significance level of 0.05, statistically strictly proving the normality of the residuals.

This series of quantitative analyses confirms the objective existence of the induced heave error from a statistical perspective, demonstrating that the method proposed in this paper can effectively identify induced heave error and provide a quantifiable decision basis for error discrimination in complex topographic scenarios.

### 4.3. Validation of the Effectiveness of the Error Correction Method

The induced heave error was corrected according to the method described in Section 3. Based on the effective matching distance, corresponding point pairs with beam incidence angles between −30° and 30° were extracted from the overlapping area of the two survey lines, resulting in 55,299 sets of valid matching data. For each corresponding point pair, the bathymetric discrepancy was calculated, and combined with the roll and pitch observations recorded by the attitude sensor at the corresponding time, the fundamental dataset for regression modeling was constructed. The three explanatory variables X, Y, Z were calculated based on Equation (7), and the VIF for each variable was calculated, with the results listed in Table 2. From Table 2, $VIF_{\max}=40,830.817>10$, indicating a severe multicollinearity problem among the variables. Therefore, the PLSR method was adopted to robustly estimate the coefficient matrix.

During the construction of the PLSR model, the 5-fold cross-validation method was used to determine the optimal number of components. The validation results showed that when the number of components was 3, the cross-validation Root Mean Square Error (RMSE) reached the minimum value of 0.233, indicating that the model has the best predictive performance and generalization ability under this configuration. Finally, the PLSR model was established based on 3 principal components, and the estimated values of the obtained regression coefficients are listed in Table 3.

**Figure 7:**
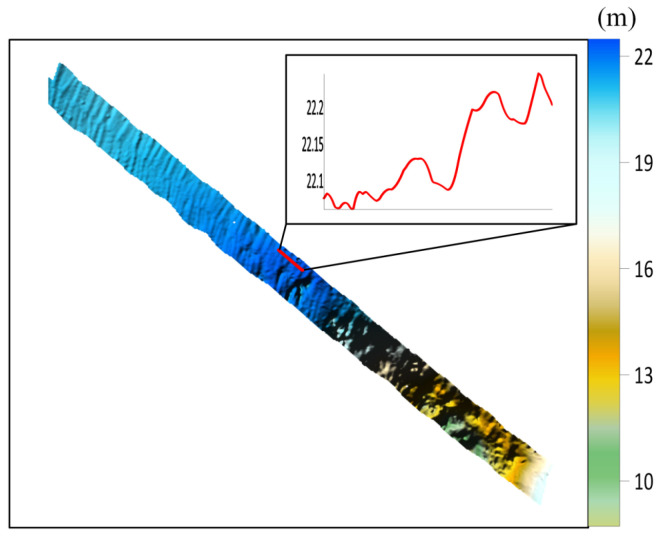
Corrected measured topographic map.

After obtaining the estimated regression coefficients, the depth values of all beam points in survey lines L1 and L2 were corrected according to Equation (24). Figure 7 shows the topographic map generated based on the corrected point cloud data. Compared with Figure 4, the original anomalous stripes along the track direction in the two survey lines have been basically eliminated after correction. The topographic profile morphology is smoother and more continuous, reflecting the undulations of the real seabed topography more reasonably. This result verifies the correctness of the earlier conclusion regarding the discrimination of induced heave error and also demonstrates the good effectiveness of the proposed error correction method in handling stripe-shaped topographic distortions caused by induced heave error.

To objectively evaluate the correction effect of the induced heave error, quantitative and qualitative analyses were conducted from two aspects: the statistics of discrepancies in the survey line overlap area and the consistency of along-track topographic profile mosaicking.

1. Status of Discrepancies in the Survey Line Overlap Area.

**Figure 8:**
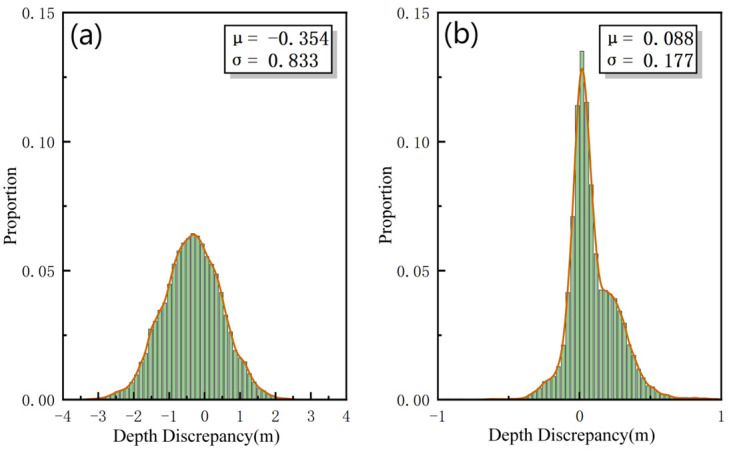
Status of discrepancies in the survey line overlap area: (**a**) The depth discrepancies before correction; (**b**) The depth discrepancies after correction.

The distribution characteristics of depth discrepancies directly reflect the consistency and accuracy level of the data. Figure 8a,b show the distribution of depth discrepancies in the overlap area of survey lines L1 and L2 before and after the induced heave error correction, respectively. Comparing Figure 8a,b, it can be seen that the histogram of the corrected discrepancies is significantly more concentrated and has a taller, narrower shape. Statistical results show that the standard deviation of the discrepancies decreased from 0.833 m before correction to 0.177 m after correction, and the mean absolute value decreased from 0.354 m to 0.088 m. This change indicates a significant reduction in systematic bias and a clear decrease in dispersion in the corrected data, verifying the effectiveness of the proposed method in improving data quality.

2. Analysis of Along-Track Profile Mosaicking for Adjacent Survey Lines.

**Figure 9:**
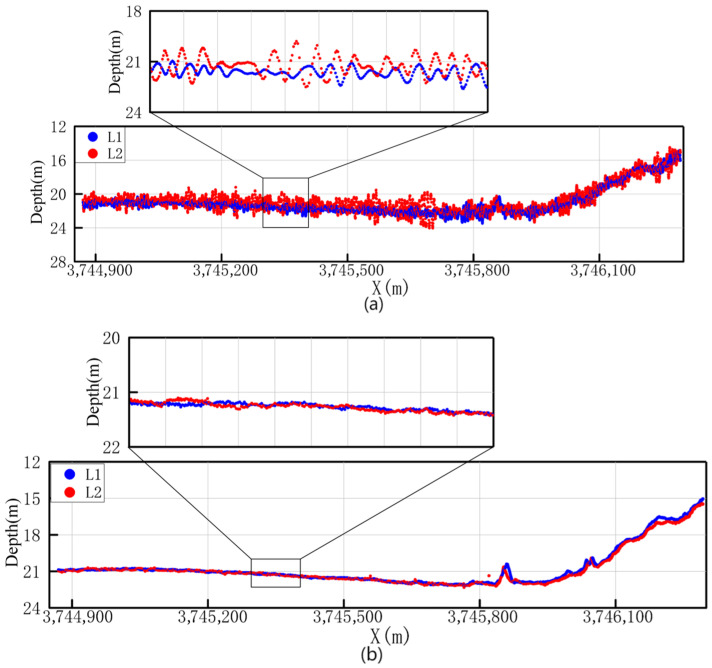
Status of along track profile mosaicking in the survey line overlap area: (**a**) The along track profile before correction; (**b**) The along track profile after correction.

To further intuitively evaluate the correction effect, profiles along the track direction were extracted and analyzed from the point cloud data before and after correction. Figure 9a,b show the topographic mosaicking situation of the two survey lines on the same along-track profile before and after correction, respectively. By comparison, it is evident that the corrected point clouds show better consistency in the profile morphology, with significantly improved mosaicking effect and enhanced topographic continuity.

In summary, the analysis above demonstrates that the method proposed in this paper can effectively suppress the impact of induced heave error on topographic data and significantly improve the accuracy and reliability of multibeam bathymetric data.

### 4.4. Reverse Verification of the Correctness of the Error Discrimination Method

The corrected data were used again for error discrimination. The new regression results and significance analysis results are shown in Table 4. The results show that the *p*-values of the *t*-test for variables X and Z are greater than 0.05, indicating that the linear driving relationship between the attitude parameters and the bathymetric discrepancy has been significantly weakened. The adjusted coefficient of determination ($adjR^{2}=0.004$) further corroborates that the attitude parameters can only explain 0.4% of the variation in bathymetric discrepancies, forming a significant contrast with the pre-correction result ($adjR^{2}=0.742$).

**Figure 10:**
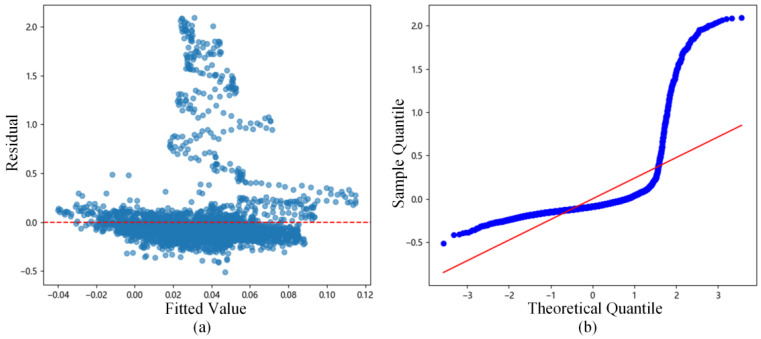
Residual analysis statistical chart for data after induced heave error correction: (**a**) Residuals vs. fitted values plot; (**b**) Residual Q-Q plot.

New residuals vs. fitted values plot (Figure 10a) and residual Q-Q plot (Figure 10b) were drawn. It can be seen in Figure 10a that the residual distribution is uneven, and in Figure 10b the points deviate severely from the diagonal. Meanwhile, the Anderson–Darling normality test statistic $A^{2}$ is 663.9711 with a p-value of 0.0000, which is below the significance level of 0.05, indicating that the residuals do not possess homoscedasticity and normality.

Based on the above analysis, it can be concluded that in the absence of induced heave error, there is no statistically significant correlation between the bathymetric discrepancies and the attitude parameters. This reversely verifies the specificity and correctness of the discrimination method proposed in this paper in capturing the error characteristics.

## 5. Conclusions

This paper systematically investigated and proposed a complete technical workflow, from discrimination to correction, targeting the induced heave error caused by attitude sensor installation biases in multibeam bathymetry. Firstly, regarding error discrimination, an objective identification method based on regression diagnostics was proposed. By establishing a multiple linear regression model between bathymetric discrepancies and attitude parameters, and comprehensively applying parameter significance testing, goodness-of-fit analysis, and tests for homoscedasticity and normality of residuals, effective detection and statistical confirmation of induced heave error were achieved. This method overcomes the difficulty of empirically distinguishing error-induced distortions from natural topographic components in complex seabed environments. Secondly, concerning error correction, the Partial Least Squares Regression (PLSR) method was introduced to address the multicollinearity problem among independent variables in the regression model. By extracting latent variables that best explain the dependent variable, PLSR constructs a stable model for estimating installation offset parameters, effectively avoiding the estimation instability of traditional least squares methods under ill-conditioned scenarios and laying a theoretical foundation for high-precision error correction. Finally, the proposed methods were tested using field survey data. The results indicate:

(1) The discrimination method based on regression diagnostics can effectively identify the presence of induced heave error with strong specificity and no misjudgment occurred, verifying the reliability of this method under complex topographic conditions.
(2) The PLSR correction method can accurately estimate the installation offset parameters of the attitude sensor. After correction, the Root Mean Square Error (RMSE) of depth discrepancies between adjacent survey lines significantly decreased from 0.833 m to 0.177 m, a reduction of 78.8%, demonstrating the notable effectiveness of this method in error suppression.
(3) Visualization of the topographic point cloud results shows that the periodic stripe-shaped distortions along the track direction were basically eliminated after correction, and the continuity of topographic profiles and the consistency of mosaicking were markedly improved. Reverse regression diagnostics performed on the corrected data further indicated that the systematic correlation between attitude parameters and depth discrepancies has been effectively removed, confirming the validity of the error correction.

In summary, the methodological framework proposed in this paper provides an effective solution for the objective identification and high-precision correction of induced heave error, offering robust technical support for enhancing the quality and reliability of multibeam bathymetric data under complex topographic conditions.

## Figures and Tables

**Table 1:** Regression results and significance analysis results for data containing induced heave error.

Variable	Fitted Coefficient	Standard Error	t-Statistic Value	*p*-Value
Intercept	0.0194	0.001	26.022	0.000
X	1.7698	0.050	35.472	0.000
Y	−2.4551	0.018	−136.679	0.000
Z	4.8543	0.600	8.094	0.000

**Table 2:** VIF values for each variable.

$\mathrm{VI}F_{X}$	$\mathrm{VI}F_{Y}$	$\mathrm{VI}F_{Z}$
40830.770	40,830.817	1.008

**Table 3:** Estimated coefficients of the PLSR model.

${\hat{\beta }}_{0}$	${\hat{\beta }}_{1}$	${\hat{\beta }}_{2}$	${\hat{\beta }}_{3}$
0.055	7.262	−7.264	−0.009

**Table 4:** Regression results and significance analysis results for data after induced heave error correction.

Variable	Fitted Coefficient	Standard Error	t-Statistic Value	*p*-Value
Intercept	0.0331	0.006	5.820	0.000
X	0.1619	0.103	1.571	0.116
Y	0.0831	0.023	3.536	0.000
Z	−0.1134	0.139	−0.815	0.415

## Data Availability

For secret, raw geo-referenced bathymetric data cannot be shared publicly.

## Abbreviations

The following abbreviations are used in this manuscript:

MBES	Multibeam Echo Sounder
PLSR	Partial Least Squares Regression
Q-Q	Quantile–Quantile
A-D	Anderson–Darling
OLS	Ordinary Least Squares
